# Integrative quantitative and qualitative analysis for the quality evaluation and monitoring of Danshen medicines from different sources using HPLC-DAD and NIR combined with chemometrics

**DOI:** 10.3389/fpls.2022.932855

**Published:** 2022-10-17

**Authors:** Qing Li, Luming Qi, Kui Zhao, Wang Ke, Tingting Li, Lina Xia

**Affiliations:** ^1^School of Chemistry, Biology and Environment, Yuxi Normal University, Yuxi, China; ^2^Chengdu Institute for Food and Drug Control, Chengdu, China; ^3^School of Health Preservation and Rehabilitation, Chengdu University of Traditional Chinese Medicine, Chengdu, China; ^4^State Administration of Traditional Chinese Medicine, Key Laboratory of Traditional Chinese Medicine Regimen and Health, Chengdu, China; ^5^College of Materials Science and Engineering, Southwest Forestry University, Kunming, China; ^6^School of Big Data and Artificial Intelligence, Chengdu Technological University, Chengdu, China

**Keywords:** *Salvia* medicines, HPLC-DAD, NIR, chemometric models, quality evaluation and monitoring

## Abstract

The root and rhizome of *Salvia miltiorrhiza* (Danshen in short) is a well-known herbal medicine used to treat cardiovascular diseases in the world. In China, the roots and rhizomes of several other *Salvia* species (Non-Danshen in short) are also used as this medicine in traditional folk medicine by local herbalists. Differences have been reported in these medicines originating from different sources, and their quality variation needs to be clearly investigated for effective clinical application. This study presented a comprehensive quality evaluation and monitoring for Danshen from 27 sampling sites and Non-Danshen from other 5 *Salvia* species based on a high-performance liquid chromatography-diode array detector (HPLC-DAD) and near-infrared (NIR), with the combination of chemometric models. The results showed that cryptotanshinone, tanshinone IIA, tanshinone I, salvianolic acid B, salvianic acid A sodium, dihydrotanshinone I, and rosmarinic acid in these medicines from different sources exhibited great variations. Referring to the standards in Chinese Pharmacopoeia (CP), European Pharmacopeia (EP), and United States Pharmacopeia (USP), Non-Danshen from *S. brachyloma, S. castanea, S. trijuga, S. bowleyana*, and *S. przewalskii* were assessed as unqualified, and Danshen in the Shandong Province had the best quality due to the high qualified rate. Based on random forest (RF) and partial least-squares discriminant analysis (PLS-DA), NIR technique could successfully monitor the quality of these medicines by discriminating the species and regions with the accuracies of 100.00 and 99.60%, respectively. Additionally, modified partial least-squares regression (MPLSR) models were successfully constructed to investigate the feasibility of NIR fingerprints for the prediction of the quality indicators in these medicines. The optimized models obtained the best results for the total of tanshinone IIA, tanshinone I, and cryptotanshinone (TTC), tanshinone IIA, and salvianolic acid B, with the relative prediction deviation (*RPD*) of 4.08, 3.92, and 2.46, respectively. In summary, this study demonstrated that HPLC-DAD and NIR techniques can complement each other and could be simultaneously applied for evaluating and monitoring the quality of Danshen medicines.

## 1. Introduction

The root and rhizome of *Salvia miltiorrhiza* (Danshen in short) is a well-known traditional Chinese medicine for the treatment of cardiovascular diseases (Li et al., 2018; Orgah et al., 2020). It is always used as a functional food and a dietary supplement in Asia, America, and Europe countries (Raposo et al., 2021). Because of its good drug efficacy and healthcare function, there is great demand for this medicine, which has led to its cultivation over increasingly wide areas. According to Ming Yi Bie Lu (an ancient pharmacological text of the Eastern Han Dynasty of China), Danshen was native to the Tongbai Valley of the Henan Province and Mount Tai of the Shandong Province, from which it gradually spread (Deng et al., 2016). Current cultivation covers a wide range of areas in China, including the provinces of Shandong, Henan, Hebei, Sichuan, Shaanxi, Anhui, Shanxi, etc. Differences have been reported in herbal medicines originating from different regions, with some areas producing better quality than others (Shahrajabian et al., 2019). In addition, a field investigation also shows the roots and rhizomes of several other *Salvia* species (Non-Danshen in short) have similar quality parameters and have been used as Danshen medicines to cure cardiovascular diseases in China (Mervić et al., 2022). In this situation, the different regions and species have been regarded as the most important factors affecting the accumulation of active ingredients which are responsible for this medicine's medicinal and healthcare quality. The comprehensive quality evaluation and monitoring study regarding different regions and species are of utmost importance for the reasonable development and utilization of Danshen medicines.

Traditional methods to monitor herbal medicine's quality, such as character identification, microscopic identification, and thin-layer chromatography identification, cannot easily evaluate the quality of these medicines from different sources due to similar appearance and chemical components (Pharmacopeia, 2015; Pharmacopoeia, 2015; Pharmacopoeia Japanese, 2017). Comparatively, a high-performance liquid chromatography-diode array detector (HPLC-DAD) which is a popular quantifiable technique can characterize the variation of a particular chemical compound in samples accurately and specifically (Liang et al., 2017; Dimcheva et al., 2019). Meanwhile, near-infrared (NIR) spectroscopy can provide a rapid, reliable, and environmentally friendly method for qualitatively collecting many descriptive metabolic profiles of samples (Zhu et al., 2018; Jiao et al., 2020; Sun et al., 2020). These methods can complement each other and have been extensively applied as the common strategy for the quantitative evaluation and qualitative monitoring of the quality of herbal medicines (Qi et al., 2018; Ni et al., 2019).

However, the collective data from HPLC-DAD and NIR instruments are always huge, which is difficult to directly analyze using simple mathematical statistical methods. Recently, a chemometric model is an effective technique to interpret the massive descriptive data and has exhibited obvious advantages compared with conventional methods. Especially, these models are playing an increasingly important role in the analysis of multi-source natural products which have a complicated metabolic profile (Deng et al., 2021). Applying chemometric models allows unsupervised visualization or supervised prediction analysis of datasets from chromatographic and spectroscopic instruments, which can more effectively achieve the quality evaluation and monitoring of herbal medicines from different sources. For example, the chemometric model of partial least squares regression (PLSR) was successfully established to analyze the correlation of chromatographic and spectroscopic data, which provided a comprehensive quality evaluation and monitoring for *Coptis* medicines from different species (Qi et al., 2018). In sum, a chemometric model can be used in the quantitative and qualitative analysis of the complex chemical system of herbal medicines and further promote the application potential of chemical sensors regarding the quality analysis of these medicines from different sources.

Thus, the present study aimed to perform a comprehensive quality evaluation and monitoring strategy for the Danshen medicines from 27 sampling sites and the Non-Danshen medicines from other 5 *Salvia* species based on HPLC-DAD and NIR techniques, with the combination of chemometric models. The main bioactive compounds, including cryptotanshinone, tanshinone IIA, tanshinone I, salvianolic acid B, salvianic acid A sodium, dihydrotanshinone I, and rosmarinic acid, were determined to evaluate their quality using the HPLC-DAD technique, respectively. Then, the qualitative data were collected using the NIR technique to present the overall metabolic variation of these medicines. Several chemometric models of t-distributed stochastic neighbor embedding (t-SNE), partial least-squares discriminant analysis (PLS-DA) and random forest (RF) were constructed to discriminate the regions and species of these medicines, respectively. Finally, modified partial least-squares regression (MPLSR) was further established to analyze the correlation of the descriptive data from HPLC-DAD and NIR techniques. According to the Chinese Pharmacopoeia (CP), the European Pharmacopoeia (EP), and the United States Pharmacopeia (USP), three quality indicators of the total of tanshinone IIA, tanshinone I, and cryptotanshinone (TTC), as well as tanshinone IIA, and salvianolic acid B were predicted to monitoring the quality of these medicines from different sources. In summary, this study can provide a comprehensive quality evaluation and monitoring for Danshen medicines from different sources, and further promote the application of chemical sensors and chemometric models for the quality analysis of multi-source herbal medicines.

## 2. Materials and methods

### 2.1. Reagents

The standard chemical components of cryptotanshinone, tanshinone IIA, tanshinone I, salvianolic acid B, salvianic acid A sodium, dihydrotanshinone I and rosmarinic acid were purchased from the National Institute for the Control of Pharmaceutical and Biological Products (Beijing, China), respectively. The purity of all standard compounds was higher than 97%. HPLC grade methanol and formic acid were purchased from Thermo Fisher Scientific (Shanghai, China). Deionized water was prepared by the Milli-Q water system (Millipore, USA). Other analytical grade reagents were supplied by Chron Chemicals Co., Ltd. (Chengdu, China).

### 2.2. Samples preparation

**Figure 1:**
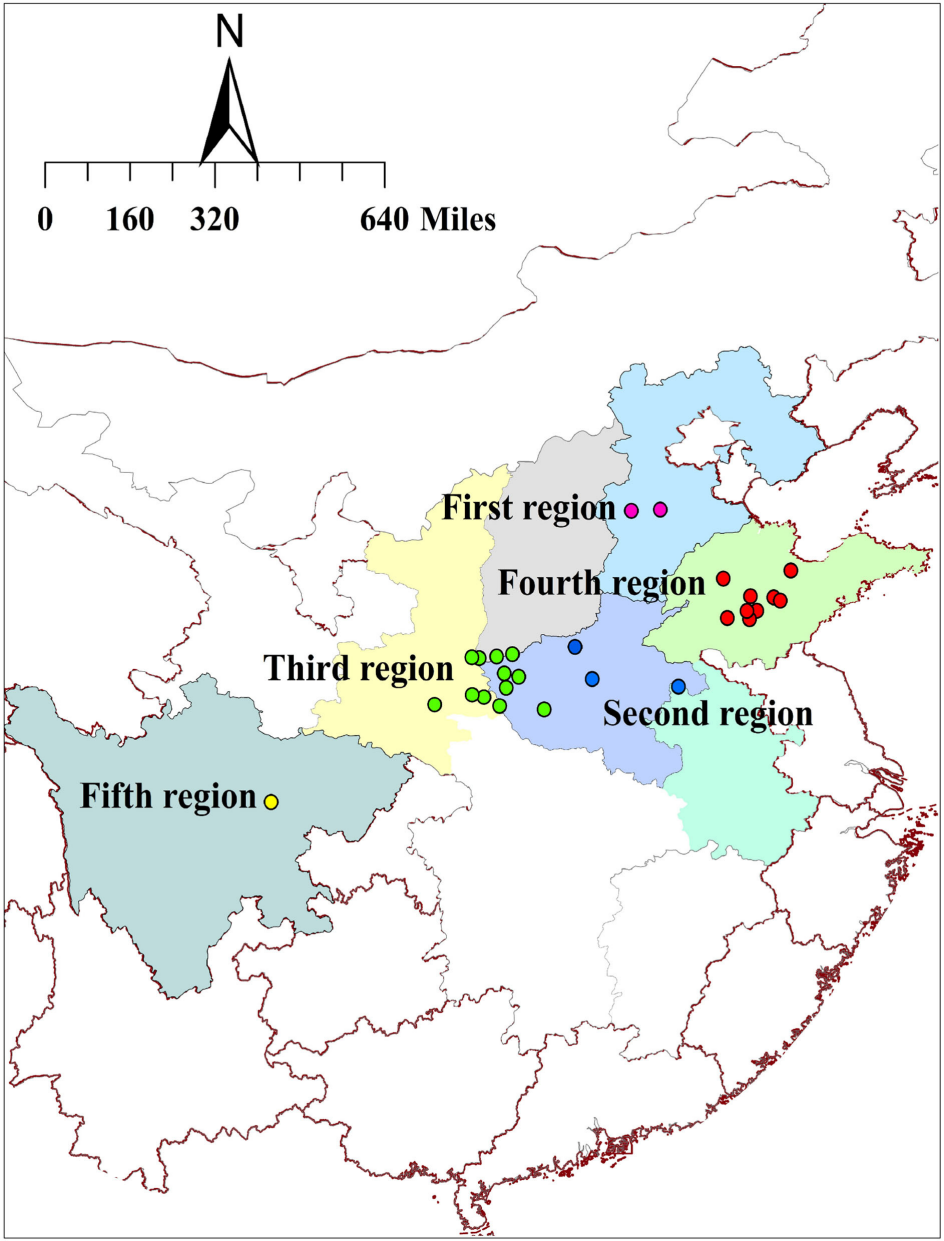
The distribution of sampling sites in 7 provinces in China. (Description: This figure shows parts of China consisting of the 27 sampling sites from 7 provinces for Danshen medicines. To obtain a better quality evaluation, we artificially divided these sites into 5 regions according to different environmental conditions).

A total of 150 samples of *S. miltiorrhiza* were collected from 27 sampling sites in 7 provinces during the harvest time. Based on the preliminary investigations, we found that regions closer to each other have more similar environmental characteristics. Hence, we artificially grouped these sampling sites based on physical distance rather than provincial administrative regions (Figure 1). In addition, the roots and rhizomes of other *Salvia* species are always used as Danshen medicines in folk applications. Other 5 *Salvia* species of *S. brachyloma, S. castanea, S. trijuga, S. bowleyana*, and *S. przewalskii* were also collected to compare their quality with Danshen medicines from *S. miltiorrhiza* species. These species were authenticated by Professor Lina Xia of Chengdu University of Traditional Chinese Medicine in China, and the remaining samples were kept in our laboratory. In order to ensure the heterogeneity of all samples, the minimum distance between the adjacent producing areas was 1 km at least. The detailed information about these materials is shown in Supplementary Tables S1–S3.

The root and rhizome of each individual were separated and washed using clean water. After cleaning and air-drying, all samples were dried in a drying oven at 60°C to a constant weight. Subsequently, these materials were smashed and sifted through an 80 mesh sieve. Finally, all of these materials were stored in a dry condition for subsequent analysis.

### 2.3. HPLC-DAD analysis

HPLC-DAD was first used to evaluate the quality of these medicines by target determining their main bioactive compounds. This technique was performed on an Agilent 1260 Series HPLC system equipped with a diode array detector, a quaternary solvent delivery system, and a column temperature controller. All samples were analyzed at a column temperature of 30°C on a Waters C18 column (150 mm × 3.9 mm, 5 μm). The mobile phases were methanol (A) and formic acid/water 0.3: 100 (v/v) (B). The gradient elution program was as follows: 0–40 min, 90–40%B; 40–50 min, 40–30%B; 50–70 min, 30-17%B; and 70–75 min, 17–90%B. The flow rate was 1.0 ml/min. The injection volume was 10 μl. The detection wavelengths were set as 330 nm for rosmarinic acid, 280 nm for salvianolic acid B, dihydrotanshinone I and salvianic acid A sodium, and 270 nm for tanshinone I, cryptotanshinone, and tanshinone IIA, respectively.

Each sample powder (0.3 g each) was weighed into clean Erlenmeyer flasks and extracted with 25 ml methanol/water 85:100 (v/v) aqueous methanol solution under ultrasonication for 30 min. After cooling, the extract solution was adjusted to its original weight by extraction solvent. The mixed standard solutions of phenolic acid compounds (salvianolic acid B, 142.59 μg/ml; rosmarinic acid, 106.41 μg/ml and salvianic acid A sodium, 11.84 μg/ml) along with tanshinone compounds (dihydrotanshinone I, 29.87 μg/ml; cryptotanshinone, 39.42 μg/ml; tanshinone I, 13.44 μg/ml; and tanshinone IIA, 55.81 μg/ml) were prepared in 85% (v/v) methanol in water. The mixture was then filtered through a 0.45 μm PVDF membrane filter for HPLC-DAD analysis. The validation results of this method are displayed in Supplementary Table S4.

### 2.4. NIR spectroscopy

Near infra red was used to monitoring the unqualified samples by discriminating their regions and species, as well as predicting the quality marking compounds of these medicines. The reflectance spectra of samples were collected on a FOSSNIRS DS-2500 spectrometer (Foss NIR Systems, Silver Spring, MD, USA) equipped with a silicon detector (850–1,100 nm) and a lead sulfide detector (1,100–2,500 nm). The samples were scanned in reflectance mode from 850 to 2,500 nm at 2 nm intervals using a cylindrical quartz ring cup (diameter 118 mm, height 38 mm). Each sample was scanned in triplicate. The mean spectrum was used for NIR analysis. Spectral data were kept in ISI scan Nova (Infra Soft International, Port Matilda, PA, USA).

Several pretreatment methods were applied using the WinISI III Project Manager (Version: 1.50, Infrasoft International, Port Matilda, PA, USA) to deal with the spectral noise signal caused by peak overlap and background interference, respectively. Smoothing was able to remove the tiny signals which were useless for the next analysis. The standard normal variable (SNV) and de-trending (DT) were used to eliminate the influence of uneven particle distribution, respectively (Samadi and Agus Arip, 2020). Derivative algorithms, including first derivative (FD) and second derivative (SD), were used to enhance the spectral resolution and eliminate baseline drift in the original NIR spectra (Roy, 2015).

### 2.5. Construction of the qualitative chemometric model

The t-SNE algorithm was employed to perform a visualization analysis of these medicines from different sources. It can transform a large set of correlated variables into several important uncorrelated components that still contain the primary information of the original variables. This algorithm is a nonlinear technique based on stochastic neighbor embedding, which transforms the similarity among data points as a probability (Anowar et al., 2021). Before model construction, visualization analysis can explore the structure of NIR spectral data and prepare for the next analysis. The t-SNE was performed using the R software (Version: 4.10, R Foundation for Statistical Computing, Vienna, Austria).

Partial least squares-discriminant analysis and RF chemometric models were comparatively established to discriminate the region and species of multi-source medicines. PLS-DA algorithm can deal with dummy variables according to the sample category and establish a model between independent and dummy variables. The algorithm determines the sample class by comparing the predicted values of each sample. A cut-off criterion is often used to assess when a sample can be considered to be correctly classified (Suhandy and Yulia, 2017). When the predicted value is higher than 0.5, the sample is defined as the correct region.

Random forest is the other algorithm for qualitative objective in this study, which has a good anti-noise ability and can effectively avoid over-fitting (Schonlau and Zou, 2020). It is based on the aggregation of multiple decision trees, and the output category was determined by the mode of the category of the individual tree output. It was generated using bootstrap samples, two-thirds of which were training samples; the rest were test samples called out-of-bag (OOB) samples used internally to get an unbiased estimate of the classification error. To establish an RF model, two important parameters, the number of decision trees (n-estimators) and the number of features (max_feature), are needed to be carefully optimized based on the OOB error estimation (Qi et al., 2020). An operational attribute of RF is its internal ability to calculate the importance of variables based on the Gini impurity index which was used to select the important variables for constructing the RF model with the best performance (Menze et al., 2009). These algorithms were performed using the R software (Version: 4.10, R Foundation for Statistical Computing, Vienna, Austria) and the SIMCA software (Version: 13.0, Umetrics, Sweden), respectively.

Confusion matrix was employed to evaluate these multi-class classification models. The indexes of true positives (*TP*), false positives (*FP*), true negatives (*TN*), and false negatives (*FN*) were calculated, and the parameters of *sensitivity, specificity*, and *accuracy* were shown for evaluating the performance of qualitative models, respectively. The formula is as follows:


(1) $$\begin{array}{l} Sensitivity=\frac{TP}{TP+FN} \end{array}$$

(2) $$\begin{array}{l} Specificity=\frac{TN}{FP+TN} \end{array}$$

(3) $$\begin{array}{l} Accuracy=\frac{TN+TP}{FP+TN+TP+FN} \end{array}$$

### 2.6. Construction of the quantitative chemometric model

The modified partial least-squares regression algorithm which is modified based on PLSR was further applied to construct the quantitative prediction models for the quality indicators of the medicines from different sources. This algorithm can be applied to extract the information related to the active components from the spectral data of herbal medicines. According to the previous papers, this method always provides better stability and accuracy than the PLSR algorithm which is well suited to handling data with high-dimensionality problems, small sample size, and multicollinearity (Yin et al., 2014; Hernandez-Jimenez et al., 2020). For this algorithm, a default 7-fold cross-validation method was used to select the optimal number of the latent variables (*LVs*) in the regression models to avoid over-fitting. The MPLSR model was constructed using the WinISI III Project Manager (Version: 1.50, Infrasoft International, Port Matilda, PA, USA).

The parameters of the coefficient of determination of calibration (${R_{c}}^{2}$), the standard error of calibration (*SEC*), and the standard error of cross-validation (*SECV*) were used to assess the effectiveness of the calibration model. The parameters of coefficient of determination of prediction ( ${R_{p}}^{2}$), the standard error of prediction (*SEP*), and ratio of residual predictive deviation (*RPD*) were applied to evaluate the prediction ability of these quantitative models.


(4) $$\begin{array}{l} \begin{array}{l} R^{2}=\frac{\sum_{i=1}^{n}{\left(O_{i}-P_{i}\right)}^{2}}{\sum_{i=1}^{n}{\left(O_{i}-O\right)}^{2}} \end{array} \end{array}$$

(5) $$\begin{array}{l} \begin{array}{l} SEC=\sqrt{\frac{\sum_{i=1}^{n_{c}}{\left(P_{i}-O_{i}\right)}^{2}}{n_{c}-1}} \end{array} \end{array}$$

(6) $$\begin{array}{l} \begin{array}{l} SECV=\sqrt{\frac{\sum_{i=1}^{n_{c}}{\left(P_{i}-O_{i}\right)}^{2}}{n_{c}}} \end{array} \end{array}$$

(7) $$\begin{array}{l} \begin{array}{l} SEP=\sqrt{\frac{\sum_{i=1}^{n_{p}}{\left(P_{i}-O_{i}\right)}^{2}}{n_{p}}} \end{array} \end{array}$$

where *P*_*i*_ is the predicted value of objective compounds in calibration or prediction set and *O*_*i*_ is the measured value in calibration or prediction set of objective compounds; *n*_*c*_ is the number of observations in the calibration set; *n*_*p*_ is the number of observations in the prediction set. The best calibrations were selected based on high ${R_{C}}^{2}$, ${R_{P}}^{2}$, and low *SECV, SEC*, and *SEP*.

Additionally, *RPD* was calculated by


(8) $$\begin{array}{l} RPD=\frac{SD}{RMSEP} \end{array}$$

where, *SD* is the standard deviation. The *RPD* value is a common statistical parameter that is used to evaluate the performance of the predicted and generalization ability of the quantitative chemometric model. The model with a high *RPD* means the better prediction performance.

**Figure 2:**
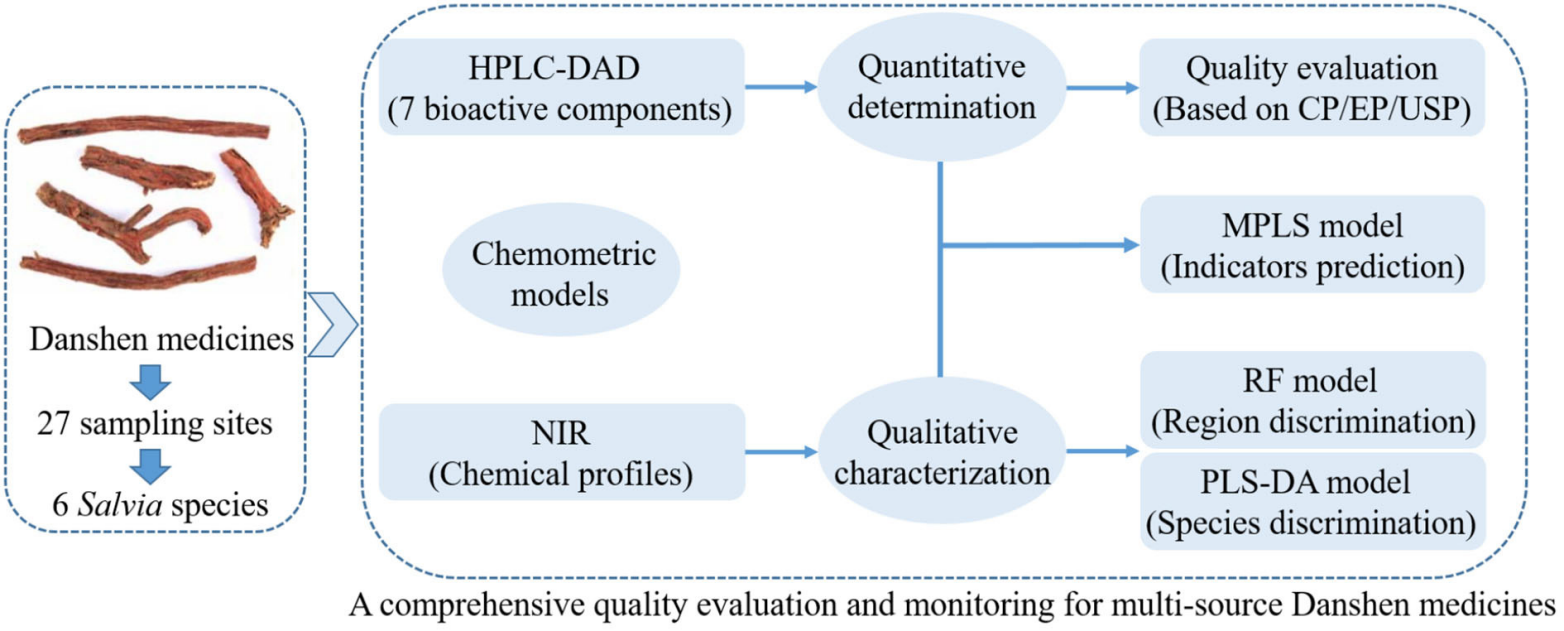
The simple data analysis flow diagram of this study. (Description: This figure shows the main steps of HPLC-DAD quantitation for active compounds, NIR fingerprint for overall metabolites, and the application of chemometric models, which complement each other for a comprehensive quality analysis for *Salvia* medicines from different sources).

To avoid over-fitting and ensure the robustness of these models, the sample set was divided into two groups using the classic Kennard-Stone selection algorithm (Kennard and Stone, 1969). Two-thirds of samples were selected as the train samples for training the calibrated model, and one-third of them were used as the test set for verifying the effectiveness of the calibrated model. A simple data flow diagram for the above steps is shown in Figure 2.

## 3. Results and discussion

### 3.1. Quantitative determination of active components

**Figure 3:**
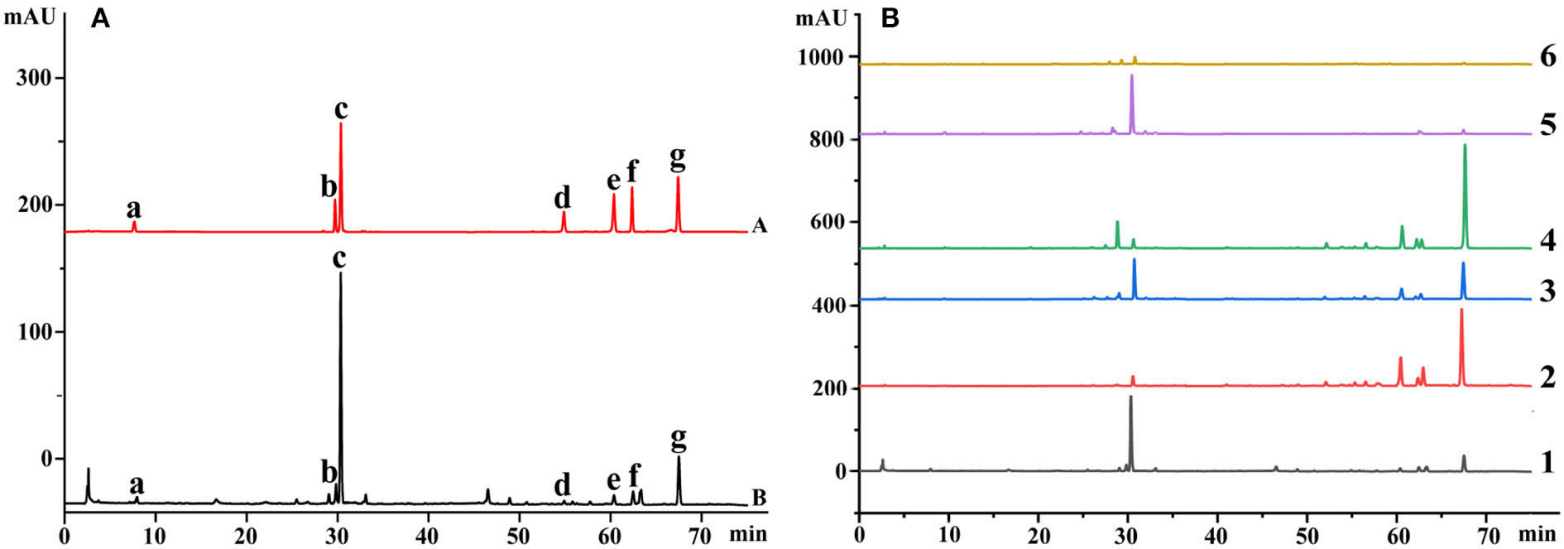
The representative chromatograms of medicines based on the HPLC-DAD technique (Description: **(A)** represents the chromatograms of mixed standard solution and Danshen medicines; **(B)** represents the chromatograms of roots and rhizomes of 6 Salvia species. The chemical compounds are salvianic acid A sodium, rosmarinic acid, salvianolic acid B, dihydrotanshinone I, cryptotanshinone, tanshinone I, and tanshinone IIA according to the retention time, respectively. The roots and rhizomes for Salvia species of 1–6 are *S. miltiorrhiza, S. trijuga, S. castanea, S. przewalskii, S. bowleyana*, and *S. brachyloma*, respectively.).

Herb medicines always contain a wide range of biological activities, and the accumulations of chemical constituents are an important target to evaluate their quality (Abate et al., 2021; Mastinu et al., 2021). To comprehensively understand the quality variation of these medicines from different sources, 7 chemical constituents representing the quality of Danshen medicines, including rosmarinic acid, salvianolic acid B, dihydrotanshinone I, salvianic acid A sodium, tanshinone I, cryptotanshinone, and tanshinone IIA, were determined using HPLC-DAD techniques, respectively. The representative chromatograms are shown in Figure 3, and the main parameters of the developed methods are listed in Table 1. As seen in Figure 3, all components are baseline separated and can be quantified accurately. The determination results are shown in Table 2.

**Table 1:** The main parameters of the developed HPLC-DAD method.

**Compounds**	**Calibration**	**Correlation**	**Linear**
	**curve**	**coefficient**	**range (μg)**
Salvianic acid A sodium	y = 0.0496x–0.2441	0.9998	0.01–0.23
Rosmarinic acid	y = 0.0145x–0.2812	0.9993	0.29–14.26
Salvianolic acid B	y = 0.0057x+0.0112	0.9996	0.21–10.64
Dihydrotanshinone I	y = 0.0083x+0.0764	0.9998	0.06–2.99
Cryptotanshinone	y = 0.0066x–0.9554	0.9990	0.08–3.94
Tanshinone I	y = 0.0037x+0.7490	0.9992	0.03–1.34
Tanshinone IIA	y = 0.0033x–0.0003	0.9999	0.11–5.58

**Table 2:** The contents of seven active components in six *Salvia* species.

**Compounds**	***S. miltiorrhiza***	***S. brachyloma***	***S. castanea***	***S. trijuga***	***S. bowleyana***	***S. przewalskii***
Salvianic acid A sodium	0.093 ± 0.079	0.067 ± 0.026	0.018 ± 0.019	0.057 ± 0.028	0.383 ± 0.095	0.022 ± 0.010
Rosmarinic acid	0.407 ± 0.204	1.048 ± 0.922	0.988 ± 0.821	0.365 ± 0.096	0.704 ± 0.153	0.227 ± 0.079
Salvianolic acid B	6.912 ± 1.798	0.517 ± 0.316	0.708 ± 0.654	2.012 ± 0.387	9.223 ± 0.577	1.779 ± 0.208
Dihydrotanshinone I	0.032 ± 0.027	0.006 ± 0.003	0.031 ± 0.020	0.142 ± 0.074	0.015 ± 0.005	0.098 ± 0.010
Cryptotanshinone	0.105 ± 0.088	0.012 ± 0.006	0.104 ± 0.111	0.455 ± 0.264	0.033 ± 0.006	0.246 ± 0.102
Tanshinone I	0.033 ± 0.027	0.013 ± 0.005	0.047 ± 0.016	0.266 ± 0.134	0.047 ± 0.004	0.233 ± 0.083
Tanshinone IIA	0.195 ± 0.118	0.039 ± 0.016	0.332 ± 0.129	1.235 ± 0.485	0.048 ± 0.021	1.269 ± 0.081

The main chemical compounds exhibited great variations in these medicines from different sources. Salvianolic acid B was identified as the most abundant compound in medicines from *S. miltiorrhiza, S. trijuga, S. bowleyana*, and *S. przewalskii*. Its highest level (9.22±0.58) was observed in the roots and rhizomes of *S. bowleyana*. Rosmarinic acid was identified as the most abundant compound in medicines from *S. brachyloma* (1.05±0.92) and *S. castanea* (0.99±0.82). In addition, roots and rhizomes of *S. trijuga* have the highest accumulations of cryptotanshinone, tanshinone IIA, tanshinone I, and dihydrotanshinone I. Salvianic acid A sodium is the highest in the roots and rhizomes of *S. bowleyana*. These constitute largely determine the healthcare and medicinal functions of these medicines. The result suggested that these medicines from different species may have an obvious quality variation.

According to the current quality evaluation standards, salvianolic acid B, cryptotanshinone, tanshinone I, and tanshinone IIA are the quality indicators of Danshen medicines (Pharmacopeia, 2015; Pharmacopoeia, 2015; Pharmacopoeia Japanese, 2017). Based on the content limits of CP (TTC≥0.25%, Salvianolic acid B≥3.0%), EP (Tanshinone IIA≥0.12%, Salvianolic acid B≥3.0%), and USP (Tanshinone IIA≥0.1%, TTC≥0.2%, and Salvianolic acid B≥3.0%), the contents of these quality indicators and qualified rates of samples from different sources are summarized in Table 3.

**Table 3:** The qualified rate of samples from different sources according to the current standards.

**Category**	**Range of content (** ***%*** **)**			**Average content (** ***%*** **)**			**Qualified rate (** ***%*** **)**		
	**TA2**	**SAB**	**TTC**	**TA2**	**SAB**	**TTC**	**CP**	**USP**	**EP**
First region	0.01–0.07	3.31–8.22	0.02–0.11	0.05	6.10	0.07	0	0	0
Second region	0.04–0.13	4.91–10.11	0.06–0.19	0.07	7.31	0.11	0	0	11
Third region	0.04–0.59	4.92–11.13	0.06–1.09	0.23	7.50	0.40	84	91	88
Fourth region	0.12–0.74	4.14–11.23	0.20–1.16	0.28	6.71	0.51	100	100	100
Fifth region	0.08–0.28	1.32–11.02	0.10–0.48	0.16	5.24	0.22	21	37	47
Other danshen	0.02–1.67	0.26–9.56	0.05–2.91	0.59	2.66	0.89	0	0	0

TA2, SAB, and TTC are the abbreviations of tanshinone IIA, salvianolic acid B and the total of tanshinone IIA, tanshinone I, and cryptotanshinone, respectively; CP, USP, and EP are the abbreviations of Chinese Pharmacopoeia,United States Pharmacopoeia, and European Pharmacopoeia, respectively. Other Danshen means these medicines from *S. brachyloma, S. castanea, S. trijuga, S. bowleyana*, and *S. przewalskii*, respectively.

First, we observed the Danshen medicines regarding five regions. The averaged contents of Salvianolic acid B are all higher than 3.0%, meaning this component in most samples from these regions is qualified and complies with pharmacopeia standards (Pharmacopeia, 2015; Pharmacopoeia, 2015; Pharmacopoeia Japanese, 2017). However, in terms of data analysis of tanshinone IIA and TTC, the overall content of samples of the fourth and third regions was at the highest and sub-optimal levels, respectively. The qualified rates of Danshen medicines in these regions are higher than 80%. The result showed the Danshen medicines in the Shandong Province have the best quality. Samples from the provinces of Shaanxi, Shanxi, and Henan are sub-optimal. The Danshen medicines from the Sichuan, An'hui, and Hebei Provinces are at a low level because their qualified rate is lower than 50%. These differences may be mainly related to the different growing environments of different regions.

In addition, the Non-Danshen medicines from other five species are unqualified according to current pharmacopeia standards. Comparatively, compounds of tanshinone IIA and TTC are high and comply with pharmacopeia standards, but their accumulations of salvianolic acid B with the averaged content of 2.66%. Therefore, the roots and rhizomes from these species may not be used as Danshen medicines in the market because the quality indicators are insufficient for the clinical application.

### 3.2. Visualization analysis

The region and species are the key factors affecting the quality of these medicines from *Salvia* genus. Next, we collected the NIR spectra fingerprints to monitor the quality of these samples. According to the conclusion of HPLC-DAD analysis, we focus on achieving two aims using chemometric models: discriminating the different regions of Danshen medicines of *S. miltiorrhiza* species; discriminating the Danshen medicines of *S. miltiorrhiza* species from Non-Danshen medicines of other *Salvia* species.

**Figure 4:**
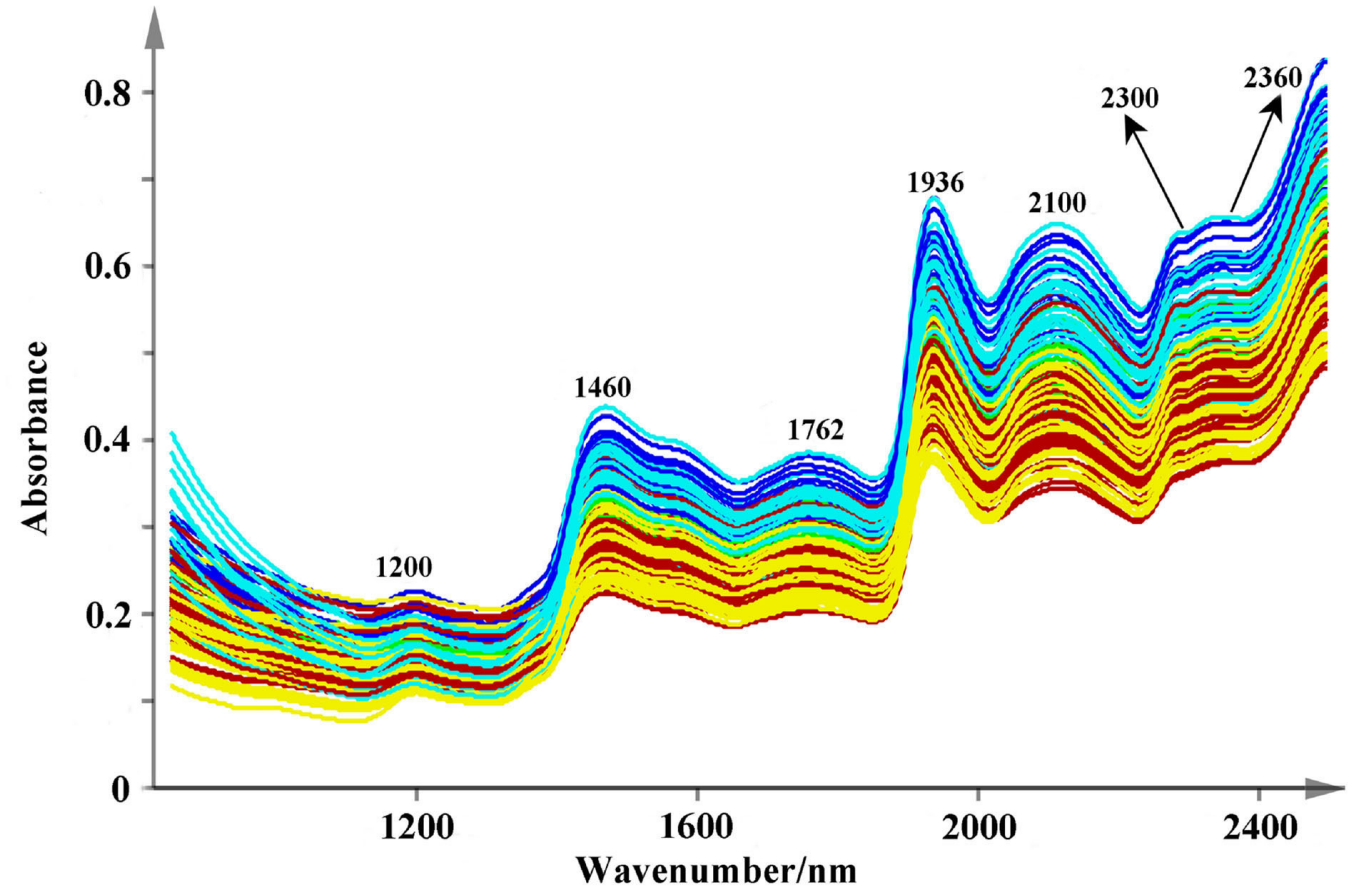
The raw NIR spectra fingerprints of Salvia medicines (Description: this figure shows the original NIR spectra of different medicines from 850 to 2,500 nm at 2 nm intervals. Several common peaks are also marked in this figure.).

**Figure 5:**
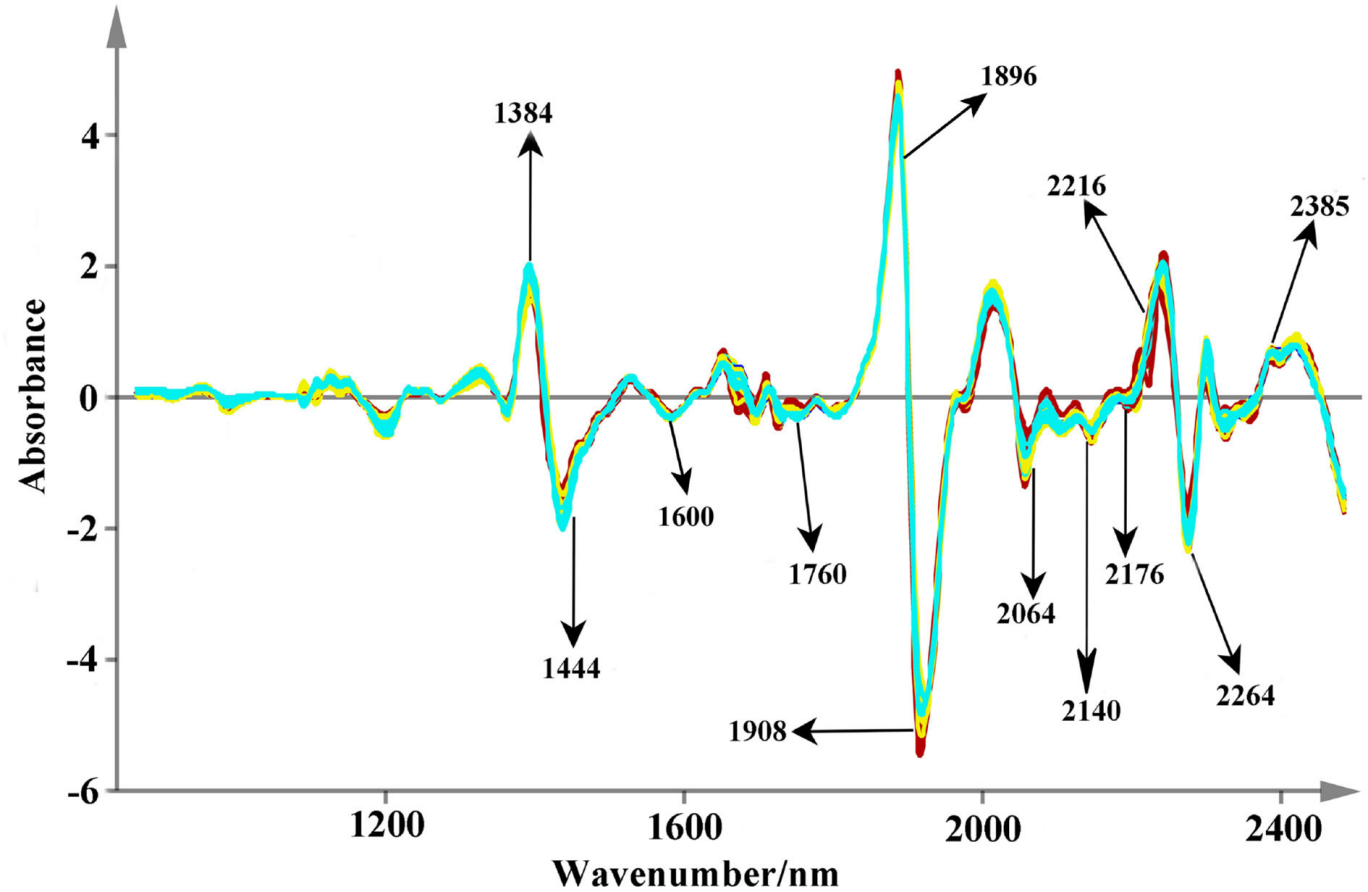
The spectra fingerprints after the pretreatments (Description: Appropriate pretreatments can improve the quality of raw spectra. In this study, the smoothing, SNV, and 2D algorithm are applied simultaneously and some valuable peaks are also marked.).

The original spectra are displayed in Figure 4. All the used NIR scans are qualified and they are located in the 99% confidence ellipses based on principal component analysis (Supplementary Figure S1). Relatively, valuable spectral peaks are mainly distributed in the range of 1,150–2,500 nm. They mainly contain the stretch or deformation vibration of C-H, O-H, and N-H groups, which are the important functional group of phenolic acids (Li and Qu, 2010). The peaks at 1,200, 1,460, 1,762, 1,936, 2,100, 2,300, and 2,360 nm are the common absorptions in the NIR spectra. Among them, a broad peak of 1,200 nm may correspond to the second over-tone of the C-H absorption band of methyl and methylene (Sun et al., 2020). Both the strong absorbance peaks around 1,460 and 1,936 nm are mainly caused by the combination of the O-H stretching vibration (Zhu et al., 2018; Gao et al., 2021). The combination bands of C-H may rise around the band of 2,300 nm (Zhu et al., 2018; Gao et al., 2021). However, due to much overlap of absorption peaks, therefore several pretreatments of smoothing, SNV, and SD were used to decompose the overlapped signs and remove the interference information (Figure 5). Peaks around 1,384 and 1,444 nm that are decomposed from 1,460 nm are related to O-H stretch first overtone and O-H bend second overtone (Toledo-Mart́ın et al., 2018). The bands in the range of 1,600–1,760 nm may be correlated with the C-H stretching, CH_2_ overtone, CH_3_ overtone, etc. (Wittkop et al., 2012; Panigrahi et al., 2016). The absorption at 1,896 nm may be due to phenolic O-H and C=O stretching second overtone (COOH) (Xiong et al., 2015). The peak around 1908 nm may correspond to the combination O-H stretch overtone (Cheng et al., 2019), which may has a correlation with the polyphenol compounds (Panigrahi et al., 2016). The N-H stretching vibration and combination bands of C=O are raised at the peaks around 2,064–2,176 nm, and the range between 2,264 and 2,385 nm may be dominated by the combination bands of C-H (Zhu et al., 2018; Sun et al., 2020). These characteristic peaks are the foundation for discriminating multi-source *Salvia* medicines.

**Figure 6:**
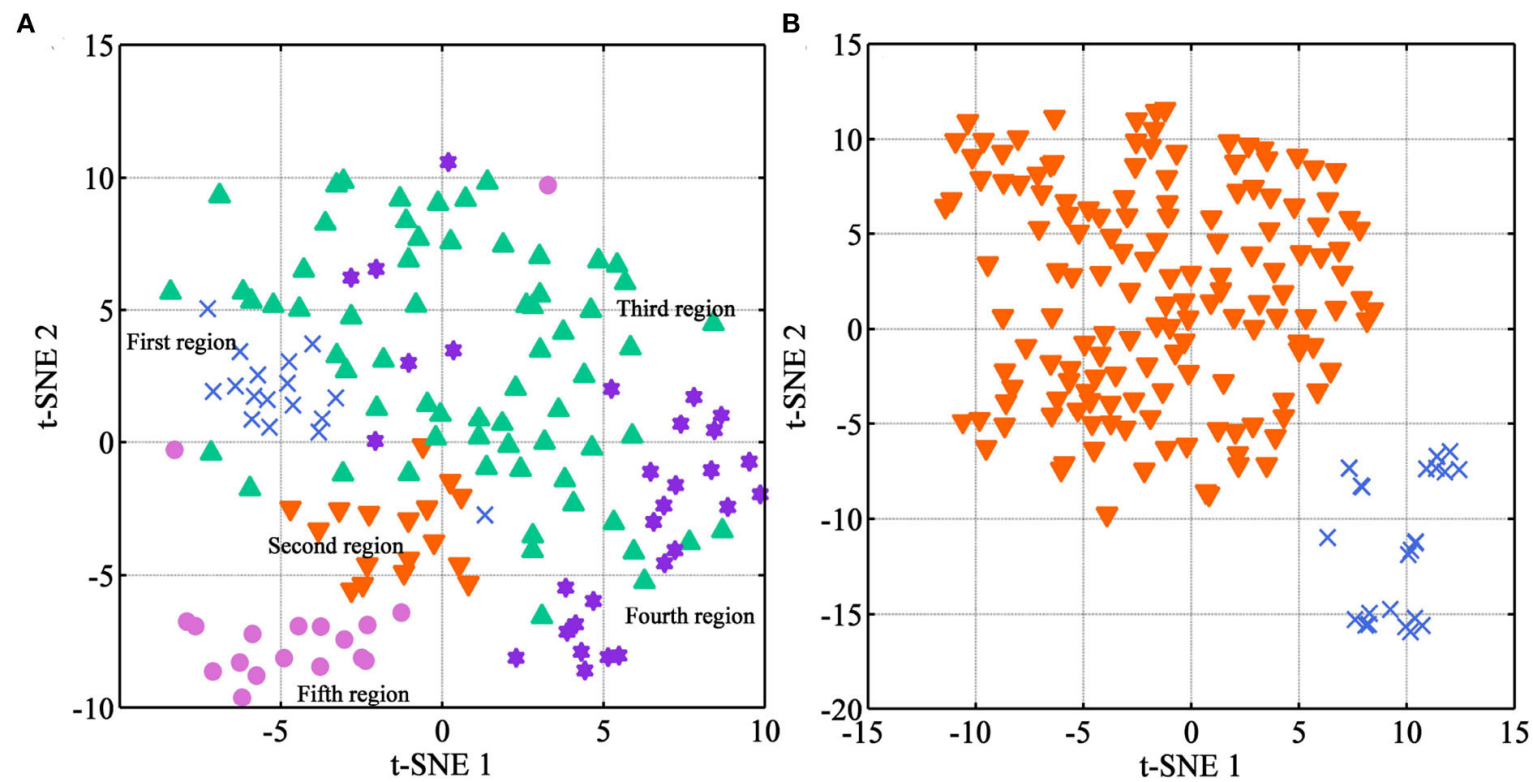
The visualization distribution of multi-source samples using t-SNE (Description: **(A)** represents the visualization result for Danshen medicines from different regions; **(B)** represents the visualization result between Danshen and Non-Danshen medicines; T-SNE 1 and t-SNE 2 represent the scores on the X and Y axes of the spectral data after dimensionality reduction, respectively.).

To better visualize the internal characteristics of these NIR data, an exploratory analysis of t-SNE was used to r visualize these medicines from different regions and species, respectively. The two-dimensional plots are shown in Figure 6. Concerning the region discrimination, the t-SNE algorithm only displays a rough separation trend for Danshen samples from five regions (Figure 6A). Overall, samples from the fourth region are near to those from the third region, which are located at the right half of the plot. Based on the HPLC-DAD analysis, medicines from these regions have the highest and sub-optimal quality, respectively. However, further analysis was necessary using the supervised algorithms.

For the samples between Danshen and Non-Danshen medicines, the result of the t-SNE algorithm is satisfactory. We can see that the medicines from *S. brachyloma, S. castanea, S. trijuga, S. bowleyana*, and *S. przewalskii* are together separated from those from *S. miltiorrhiza* species. Our result demonstrated that Danshen medicines from *S. miltiorrhiza* species had an apparent variation with Non-Danshen from other *Salvia* species from an overall qualitative point of view. Based on the HPLC-DAD conclusion, the Non-Danshen medicines from *S. brachyloma, S. castanea, S. trijuga, S. bowleyana*, and *S. przewalskii* are unqualified according to pharmacopeia standards. T-SNE algorithm can well separate Danshen of *S. miltiorrhiza* from the Non-Danshen of other *Salvia* species.

### 3.3. Qualitative models for discriminating the region and species

According to HPLC-DAD analysis, the quality and qualification rate of medicines greatly varied with different regions and species. To a certain extent, the unsupervised visualization model was powerless. PLS-DA and RF algorithms were further comparatively applied for the qualitative model to discriminate the regions and species for monitoring the medicines from different sources.

For the region discrimination, the results of these models are shown in Table 4. Regarding to the PLS-DA model, first 5 *LVs* representing 81% cumulative interpretation ability were selected to build the qualitative model for avoiding model over-fitting. According to the confusion matrix, the averaged *sensitivity, specificity*, and *accuracy* of the developed model for the test set are 85.82, 97.60, and 96.00%, respectively. Comparatively, *sensitivity* of the first and fourth regions is unsatisfactory, which is lower than 80%. Four samples of these regions are misidentified into the third region. The discrimination performance can be further improved by other more advanced algorithms. Therefore, we focused on applying the RF algorithm to promote the accuracy of region discrimination for Danshen medicines.

**Table 4:** The performance of chemometric models for the region discrimination based on NIR spectra.

**Regions**	**Models**	**Parameters**	***Sensitivity***	***Specificity***	***Accuracy***
Region 1	PLS-DA	*LV*=5	0.60	1.00	0.96
Region 2			1.00	0.98	0.98
Region 3			0.91	0.93	0.92
Region 4			0.78	0.98	0.94
Region 5			1.00	1.00	1.00
Region 1	RF	N_estimators=745	1.00	1.00	1.00
Region 2		Max_feature as=26	0.80	1.00	0.98
Region 3			0.87	0.96	0.92
Region 4			1.00	0.93	0.94
Region 5			1.00	1.00	1.00
Region 1	RF	N_estimators=745	1.00	1.00	1.00
Region 2	(Important variables)	Max_feature as=27	1.00	1.00	1.00
Region 3			0.96	1.00	0.98
Region 4			1.00	1.00	1.00
Region 5			1.00	1.00	1.00

**Figure 7:**
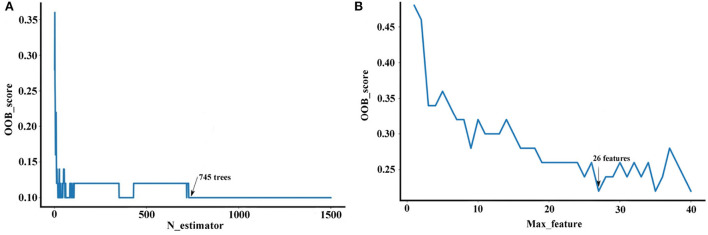
The parameter selection of the RF model (Description: **(A)** represents the parameter selection approaches for the n-estimator; **(B)** represents the parameter selection approaches for max_feature. These parameter selection were performed according to OOB error, which can guarantee an excellent predicted and generalization ability for the developed RF model.).

Random forest method is an ensemble learning technique that combines a certain number of tree classifiers, which are not related to each other. The number of tree classifiers is defined as n_estimator and the number of feature subset is defined as max_feature. The two parameters are important to balance the prediction performance and practicability of the developed RF model. Based on the NIR data of Danshen medicines, the ranges of n_estimators and max_features were initially set as 0–1,500 and 0–40, respectively. Then, these parameters were evaluated according to the minimization of OOB error (Figure 7). Optimizing the n_estimators and max_feature as 745 and 26 can achieve the best RF model with a short training time and a stable performance for the region discrimination based on the NIR dataset. As seen in Table 4, the RF model obtains better performance than the PLS-DA model, with the averaged *sensitivity, specificity*, and *accuracy* for the test set of 93.39, 97.80, and 96.80%, respectively. Particularly, for the misidentified samples in the first and fourth regions based on the PLS-DA model, the *sensitivity* of these regions can be improved to 100.00%.

**Figure 8:**
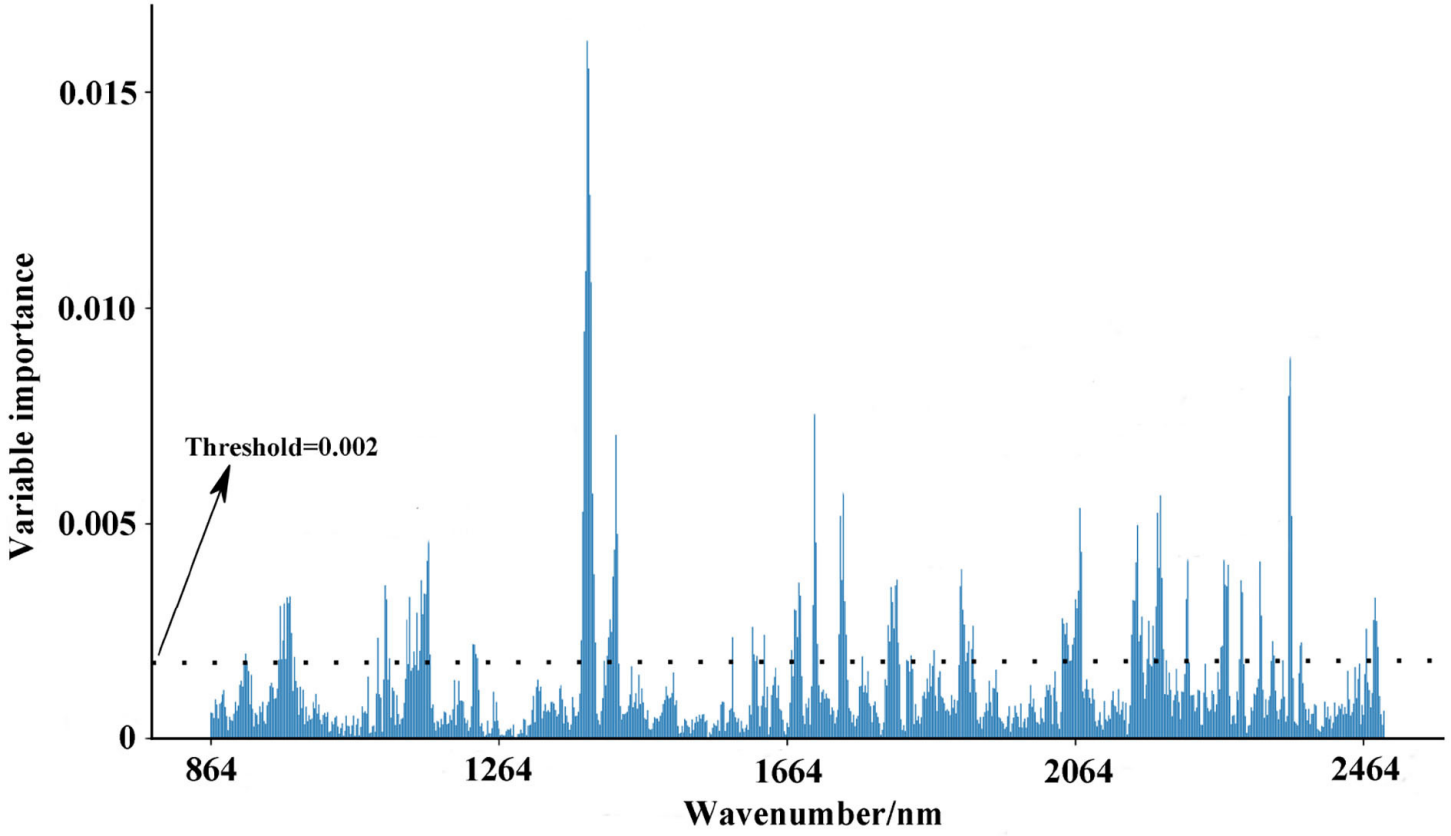
The variable importance of NIR variables based on the Gini impurity index (Description: this figure displays the variable evaluation procedure after parameter selection. After the elimination of unimportant variables, the accuracy of the RF model can be further improved.).

Further, we used the Gini impurity index to calculate the importance of NIR variables, in order to apply the variables with large importance as feature variables for modeling optimal region discrimination of Danshen medicines. The result is displayed in Figure 8. Each variable has different importance, with values ranging between 0.00001 and 0.01596, indicating that each variable has a different contribution to region prediction in the RF model. We gradually increased the threshold value in 0.001 increments to eliminate unimportant variables from the model and found the optimal threshold value corresponding to the highest accuracy of the model. When the threshold is set as 0.002, invalid variables can be removed to the greatest extent and restructure a more effective RF model. As seen in Table 4, the restructure model obtained the best discrimination performance with the averaged *sensitivity, specificity*, and *accuracy* for the test set of 99.13, 100.00, and 99.60%, respectively. As previously described, Danshen medicines from the first and second regions are of relatively poor quality, so it is very important to track their regions accurately. The restructuring model RF model can track the samples from these regions with a 100.00% accuracy. According to the confusion matrix, only one sample belonging to the third region was misclassified into the second region. Based on variable selection, the performance of the RF algorithm was further improved, demonstrating that NIR spectroscopy can provide a rapid and simple tool for the discrimination of Danshen medicines originating from different regions.

**Figure 9:**
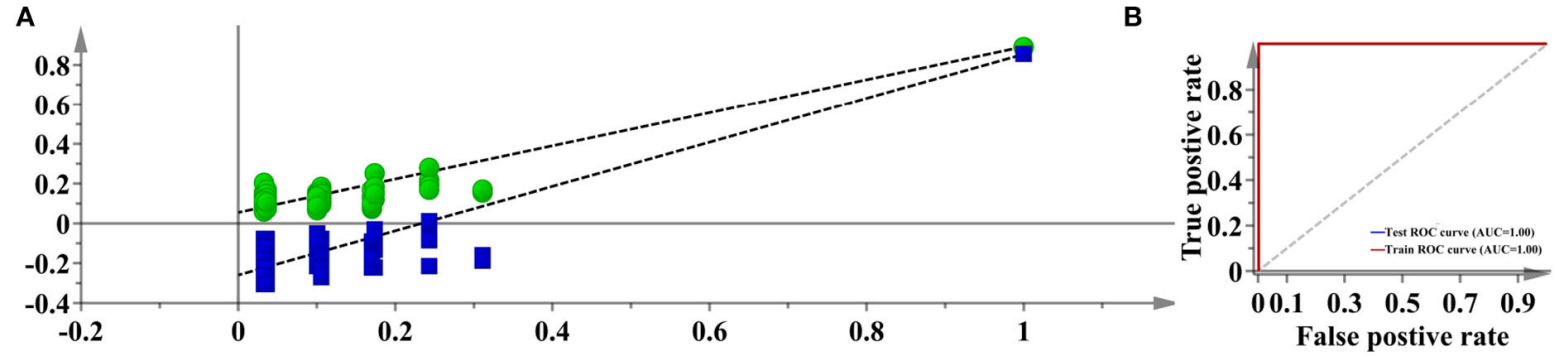
The permutation and AUC plots of the developed PLS-DA model (Description: **(A)** represents the permutation plot; **(B)** represents the AUC plot. According to the visualization result, PLS-DA confidently exports a 100% accuracy for discriminating between Danshen and Non-Danshen medicines. We show the AUC and permutation plots to indicate the reliability of this model.).

With regard to the species discrimination, the t-SNE algorithm has achieved an excellent separation. We further used supervised PLS-DA to predict whether unknown samples can be accurately classified or not. According to the 7-fold cross-validation, the first 3 *LVs* which represent 89.20% accumulated variation of the original dataset were used to construct the PLS-DA model. The AUC and permutation plots were given in Figure 9, indicating that the developed model was highly reliable. The averaged *sensitivity, specificity*, and *accuracy* for the unknown samples are 100.00%, respectively. This result showed that the PLS-DA could be fully competent for the discrimination between Danshen and Non-Danshen medicines, and therefore the RF model was not used further.

### 3.4. Quantitative models for the predicting quality indicators

The determination of quality indicators, which is always achieved using the chromatographic methods, is one of the main strategies for the quality evaluation of traditional Chinese medicines. However, the chromatographic methods involve complex and time-consuming pretreatment processes with the consumption of solvents. In recent years, with the development of chemometric models, NIR spectroscopy has been demonstrated as a more rapid and green approach for the quantitative task regarding the quality indicators of herbal medicines. Therefore, the MPLSR model was applied to construct the quantitative models to determine the quality indicators of multi-source medicines.

For the quantitative models, NIR dataset was used to construct the calibrated models and HPLC-DAD data was used to validate their accuracy and generalization ability of the developed model. Because the spectral scans in the range of 850–1,100 nm are relatively weak, these signal may be useless for the chemometric models. Hence, we comparatively applied three band ranges of 850–1,100 nm, 1,100–2,500 nm, and 850–2,500 nm, as well as four pretreatments of FD, SD, SNV, and DT to proposed the best MPLS quantitative models for the prediction of tanshinone IIA, salvianolic acid B, and TTC, which are the main quality indicators in CP, EP, and USP.

The results regarding the MPLS quantitative models are shown in Table 5 and Supplementary Table S5. The range of 1,100–2,500 nm of NIR spectra can obtain the best performance for the prediction models of three quality indicators. After the comparison, the combined application of SD, SNV, and DT pretreatments performed the best MPLSR quantitative models for Tanshinone IIA and TTC determinations. Twelve *LVs* were selected to construct the quantitative model for determining tanshinone IIA, which showed the highest ${R_{c}}^{2}$ and ${R_{p}}^{2}$ of 0.933 and 0.932, and the lowest *SEC, SECV*, and *SEP* of 0.023, 0.029 and 0.025, respectively. With respect to TTC quantitative model, 14 *LVs* were selected and the evaluated parameters of ${R_{c}}^{2}$, ${R_{p}}^{2}$, *SEC, SECV*, and *SEP* are 0.944, 0.941, 0.043, 0.045, and 0.040, respectively. Additionally, FD, SNV, and DT were the best pretreatments for the quantitative model construction to predict the content of salvianolic acid B. 14 *LVs* were applied to construct the quantitative model, with the highest ${R_{c}}^{2}$ and ${R_{p}}^{2}$ of 0.906 and 0.835, and the lowest of *SEC, SECV*, and *SEP* of 0.513, 0.556, and 0.723, respectively.

**Table 5:** The MPLS models for the quantitative determination of three quality indicators.

**Model**	**Wavelength**	**Pretreatments**	***LV***	*** ${R_{c}}^{2}$ ***	***SEC***	***SECV***	*** ${R_{p}}^{2}$ ***	***SEP* (*%*)**	***RPD***
TA2	1,100–2,500	SD; SNV; DT	12	0.933	0.023	0.029	0.932	0.025	3.92
SAB	1,100–2,500	FD; SNV; DT	14	0.906	0.513	0.556	0.835	0.723	2.46
TTC	1,100–2,500	SD; SNV; DT	14	0.944	0.043	0.045	0.941	0.040	4.08

TA2, SAB, and TTC are the abbreviations of tanshinone IIA, salvianolic acid B, and the total of tanshinone IIA, tanshinone I, and cryptotanshinone, respectively.

*Relative prediction deviation* is a commonly used parameter for comprehensively assessing the effectiveness of the model's generalization ability (Qi et al., 2017). In general, a quantitative model with an *RPD* value higher than 2 may be considered to be reliable, and an *RPD* value greater than 3 was believed to be an excellent prediction performance for unknown samples (Feng et al., 2019). In this study, TTC determination obtained the best prediction accuracy with the highest *RPD* value of 4.08. The *RPD* value of the quantitative model for tanshinone IIA determination was 3.92, indicating that the generalization effectiveness of this model was also excellent. Comparatively, the quantitative model of salvianolic acid B was relatively weaker, with an *RPD* value of 2.46, but this result can be acceptable for determining this quality indicator.

**Figure 10:**
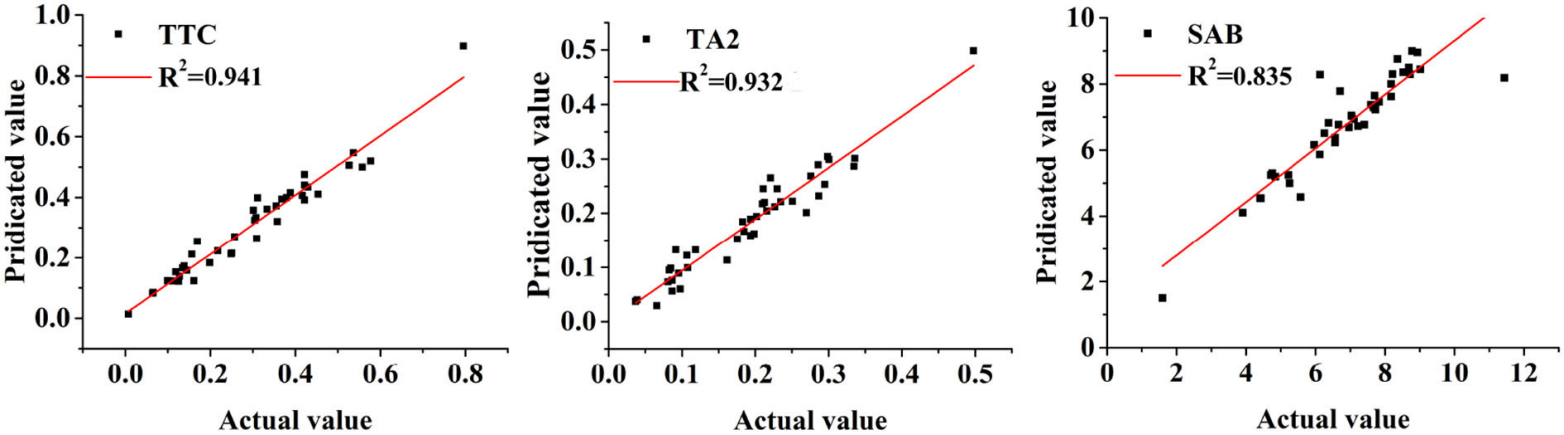
The correlation diagrams of the NIR predicted and HPLC-DAD data regarding three quality indicators (Description: TTC represents the total of tanshinone IIA, tanshinone I, and cryptotanshinone; TA2 and SAB represents the compounds of tanshinone IIA and salvianolic acid B, respectively. As seen from this figure, these observations are evenly distributed on both sides of the correlation equation, indicating these models are effective to predict these quality indicators.).

To further confirm the effectiveness of developed quantitative models, the predicted contents of three quality indicators using these MPLSR models were compared with their real concentrations based on the HPLC-DAD method in correlation diagrams. The plots are shown in Figure 10. As seen in these plots, the predicted values are much correlated with the standard concentrations, indicating these quantitative models were reliable and robust for the determination of these three quality indicators.

## 4. Conclusion

The medicinal quality of herbal medicines is difficult to control because they always come from multiple sources caused by many factors such as regions and species. In addition, they are always complex mixtures with a variety of chemical compositions, which are difficult to characterize based on a single technique comprehensively.

Here is reported a comprehensive quality evaluation and monitoring of Danshen medicines from 27 sampling sites and Non-Danshen medicines from other 5 *Salvia* species using HPLC-DAD and NIR techniques, with the combination of chemometric models. The main active components that represent the medicinal and healthcare properties were quantified, respectively, demonstrating that Danshen medicines from the fourth region (sampling sites in the Shandong Province) have the best quality due to the high qualified rate, while those in the first region (sampling sites in the Heibei Province) and the second region (sampling sites in the central and western An'hui Province) are at a low level. Non-Danshen medicines from *S. brachyloma, S. castanea, S. trijuga, S. bowleyana*, and *S. przewalskii* cannot replace Danshen medicines according to the standards in CP, EP, and USP. To monitor the quality variation of multi-source medicines, the RF models were successfully constructed to discriminate the regions of Danshen samples, with 99.60% accuracy in the test set, and the PLS-DA models effectively achieved the discrimination between Danshen and Non-Danshen medicines, with 100.00% accuracy for the test set, respectively. In addition, regarding three quality indicators of tanshinone IIA, salvianolic acid B, and TTC, the MPLSR quantitative models were successfully optimized and developed with the *RPD* of 3.92, 2.46, and 4.08, respectively.

In sum, we presented an integrative quantitative and qualitative analysis for the quality evaluation and monitoring of multi-source *Salvia* medicines from different regions and species using HPLC-DAD and NIR combined with chemometrics. The quality variation of these multi-source medicines has been effectively characterized at the quantitative and qualitative levels, which is beneficial for the reasonable development and utilization of Danshen medicines in China. The chemical sensors of HPLC-DAD and NIR combined chemometric models can be worth generalizing for the quality assessment of other multi-source herbal medicines.

## Data availability statement

The original contributions presented in the study are included in the article/Supplementary material, further inquiries can be directed to the corresponding authors.

## Author contributions

QL and LQ conceived and designed the research and wrote the manuscript. KZ, WK, and TL performed the experiments. LX revised the manuscript. All authors contributed to this article and approved the submitted version.

## Funding

This work were financially supported by the National Natural Science Foundation of China (81774387).

## Conflict of interest

The authors declare that the research was conducted in the absence of any commercial or financial relationships that could be construed as a potential conflict of interest.

## Publisher's note

All claims expressed in this article are solely those of the authors and do not necessarily represent those of their affiliated organizations, or those of the publisher, the editors and the reviewers. Any product that may be evaluated in this article, or claim that may be made by its manufacturer, is not guaranteed or endorsed by the publisher.

## Abbreviations

NIR: Near-infrared
HPLC-DAD: High-performance liquid chromatography-diode array detector
RF: Random forest
PLS-DA: Partial least-squares discriminant analysis
MPLS: Modified partial least-squares regression
PLSR: partial least squares regression
TTC, The total of tanshinone IIA: tanshinone I and cryptotanshinone
CP: Chinese Pharmacopoeia
EP: European Pharmacopoeia
USP: United States Pharmacopeia
PCA: principal component analysis
DT: De-trending
SNV: Standard normal variable
FD: First derivative
SD: Second derivative
T-SNE: T-distributed stochastic neighbor embedding
OOB: Out-of bag
*LV*: Latent variable
${R_{c}}^{2}$: Coefficient of determination of calibration
${R_{p}}^{2}$: Coefficient of determination of prediction
*SEC*: Standard error of calibration
*SECV*: Standard error of cross-validation
*SEP*, Standard error of prediction *RPD*: Relative prediction deviation.
