# Supplementary material for: Integrative quantitative and qualitative analysis for the quality evaluation and monitoring of Danshen medicines from different sources using HPLC-DAD and NIR combined with chemometrics
Source: Front Plant Sci. 2022 Oct 17;13:932855. doi: 10.3389/fpls.2022.932855 (PMC9618615; doi:10.3389/fpls.2022.932855)
Supplement: Supplementary file 1 [file Data_Sheet_1.docx]

**Table S1. The detail information of samples in this study**

| Category | Quantity | Soil Type | Landform | Description |
| --- | --- | --- | --- | --- |
| First region | 17 | Sandy | Plain | 2 sampling points of the Hebei Province |
| Second region | 17 | Sandy | Plain | 2 sampling points of Henan Province;1 sampling points of the An’hui Province |
| Third region | 67 | Yellow brown | Hill | 5 sampling points of the Shaanxi Province; 3 sampling points of the Shanxi Province; 4 sampling points of the Henan Province |
| Fourth region | 30 | Yellow brown | Hill | 9 sampling points of the Shandong Province |
| Fifth region | 19 | Yellow brown | Hill | 1 sampling points of the Sichuan Province |
| Other Danshen | 25 | Yellow brown; Red brown | Highland | 2 sampling points of the Sichuan Province; 1 sampling points of the Jiangxi Province; 1 sampling points of the Yunnan Province |

(Note: This table presents the sampling information of Danshen medicines from 6 *Salvia* species, which contains the number of samples, soil type, landform and specific sampling locations.)

**Table S2: The specific sampling sites and related environmental factors for *S. miltiorrhiza***

| Mark | Sampling sites | Longitude | Latitude | Annual average  temperature | Average annual precipitation | Average precipitation  in October (mm) | Average temperature  in October (°C) | Average high temperature in October (°C) | Average minimum temperature in October (°C) | Solar radiation  in october (kJ/m2/day) | Altitude  (m) |
| --- | --- | --- | --- | --- | --- | --- | --- | --- | --- | --- | --- |
|  |  |  |  |  |  |  |  |  |  |  |  |
|  |  |  |  |  |  |  |  |  |  |  |  |
| Pink dot | Tang County, Heibei Province | 114.4867 | 38.3965 | 12.75 | 467 | 20 | 13.9 | 20.70 | 7.1 | 13497 | 113 |
| Pink dot | Anguo Country, Heibei Province | 115.2793 | 38.4233 | 13.08 | 433 | 12 | 14 | 20.40 | 7.6 | 13386 | 33 |
| Blue dot | Wen Country, Henan Province | 112.9511 | 34.9573 | 14.42 | 570 | 41 | 15.6 | 21.20 | 9.9 | 12764 | 114 |
| Blue dot | Yuzhou County, Henan Province | 113.4197 | 34.144 | 14.85 | 696 | 49 | 16 | 21.80 | 10.2 | 12644 | 125 |
| Blue dot | Qiaocheng County, Anhui Province | 115.7725 | 33.9521 | 14.80 | 775 | 48 | 15.9 | 21.60 | 10.3 | 13944 | 40 |
| Green dot | Zhen'an County, Shaanxi Province | 109.1201 | 33.4905 | 11.56 | 775 | 77 | 11.9 | 16.30 | 7.5 | 11064 | 994 |
| Green dot | Dali County, Shaanxi Province | 110.1387 | 34.6957 | 14.09 | 581 | 57 | 14.6 | 20.30 | 8.8 | 12030 | 332 |
| Green dot | Aoli County, Shanxi Province | 110.3383 | 34.6719 | 13.63 | 588 | 55 | 14 | 19.60 | 8.5 | 12107 | 515 |
| Green dot | Donglu County, Shanxi Province | 110.8127 | 34.7149 | 13.46 | 582 | 53 | 13.9 | 19.70 | 8.1 | 12196 | 532 |
| Green dot | Hubin County, Henan Province | 111.2431 | 34.7749 | 13.80 | 578 | 50 | 14.2 | 19.80 | 8.7 | 12341 | 460 |
| Green dot | Lingbao County, Henan Province | 111.0181 | 34.2891 | 12.02 | 659 | 59 | 12.6 | 18.50 | 6.8 | 12097 | 927 |
| Green dot | Luoning County, Henan Province | 111.4141 | 34.2037 | 11.95 | 690 | 60 | 12.7 | 18.00 | 7.3 | 12098 | 935 |
| Green dot | Lushi County, Henan Province | 111.0725 | 33.9247 | 12.18 | 732 | 66 | 12.9 | 18.10 | 7.6 | 11921 | 1075 |
| Green dot | Shangzhou County, Shaanxi Province | 110.1457 | 33.7441 | 13.94 | 734 | 72 | 14.4 | 19.60 | 9.2 | 11569 | 628 |
| Green dot | Danfeng County, Shaanxi Province | 110.4683 | 33.6811 | 12.50 | 780 | 72 | 13.1 | 17.80 | 8.3 | 11644 | 1092 |
| Green dot | Shangnan County, Shaanxi Province | 110.8925 | 33.4536 | 14.83 | 790 | 75 | 15.4 | 20.70 | 10.2 | 11583 | 577 |
| Green dot | Nanzhou County, Henan Province | 112.1105 | 33.3629 | 12.43 | 876 | 71 | 13.2 | 18.30 | 8.2 | 11815 | 929 |
| Red dot | Tianqiao County, Shandong Province | 116.9985 | 36.6809 | 14.06 | 755 | 38 | 15.4 | 20.00 | 10.7 | 13954 | 28 |
| Red dot | Shouguang County, Shandong Province | 118.8415 | 36.8853 | 13.18 | 673 | 33 | 15 | 20.90 | 9 | 14026 | 16 |
| Red dot | Qufu County, Shandong Province | 117.1055 | 35.6881 | 13.43 | 708 | 33 | 15 | 20.90 | 9 | 14268 | 101 |
| Red dot | Laiwu County, Shandong Province | 117.7369 | 36.2333 | 12.84 | 727 | 32 | 14.2 | 20.00 | 8.4 | 14214 | 220 |
| Red dot | Yiyuan County, Shandong Province | 118.3705 | 36.2047 | 11.86 | 759 | 32 | 13.4 | 19.30 | 7.5 | 14177 | 373 |
| Red dot | Yiyuan County, Shandong Province | 118.5517 | 36.1241 | 11.68 | 780 | 33 | 13.4 | 19.40 | 7.3 | 14143 | 386 |
| Red dot | Xintai County, Shandong Province | 117.6381 | 35.8659 | 12.96 | 752 | 31 | 14.4 | 20.00 | 8.9 | 14274 | 221 |
| Red dot | Mengyin County, Shandong Province | 117.9133 | 35.8689 | 12.46 | 765 | 31 | 13.9 | 20.00 | 7.9 | 14237 | 298 |
| Red dot | Xintai County, Shandong Province | 117.6381 | 35.8659 | 12.96 | 752 | 31 | 14.4 | 20.00 | 8.9 | 14274 | 221 |
| Yellow dot | Zhongjiang County, Sichuan Province | 104.6617 | 31.0275 | 16.61 | 975 | 47 | 17.5 | 20.70 | 14.2 | 8299 | 445 |

(Note: This table presents the detail sampling information of Danshen medicines from *S. miltiorrhiza* species, which contains temperature, altitude, precipitation and specific sampling locations.)

**Table S3: The specific sampling sites and related environmental factors for other species**

| Species | Sampling sites | Longitude | Latitude | annual average  temperature | Average annual precipitation | Average precipitation  in October (mm) | Average temperature  in October (°C) | Average high temperature in October (°C) | Average minimum temperature in October (°C) | Solar radiation  in october (kJ/m2/day) | Altitude  (m) |
| --- | --- | --- | --- | --- | --- | --- | --- | --- | --- | --- | --- |
|  |  |  |  |  |  |  |  |  |  |  |  |
|  |  |  |  |  |  |  |  |  |  |  |  |
| *S. brachyloma* | Muli County, Sichuan Province | 101.0225 | 27.95583 | 14.23 | 789 | 60 | 14.8 | 19.5 | 10 | 11660 | 2072 |
| *S. castanea* | Muli County, Sichuan Province | 100.9278 | 28.64333 | 5.87 | 760 | 53 | 7.1 | 12 | 2.1 | 11730 | 3651 |
| *S. trijuga* | Muli County, Sichuan Province | 101.0325 | 28.3025 | 4.17 | 784 | 58 | 5.3 | 10.4 | 0.2 | 11566 | 3861 |
| *S. bowleyana* | Yihuang County, Jiangxi Province | 116.1464 | 27.49139 | 16.86 | 1743 | 74 | 18.4 | 22.6 | 14.2 | 14096 | 374 |
| *S. przewalskii* | Deqin County, Yunnan Province | 98.95141 | 28.48024 | -0.47 | 664 | 52 | 1.5 | 6.9 | -4 | 12997 | 4649 |

(Note: This table presents the detail sampling information of Non-Danshen medicines from other 5 *Salvia* species, which contains temperature, altitude, precipitation and specific sampling locations.)

**Table S4. The method validation of developed HPLC-DAD method**

| Compounds | Precision(n=6) | Stability (n=6) | Repeatability (n=6) | Recovery (n=3) | |
| --- | --- | --- | --- | --- | --- |
|  | RSD (%) | RSD (%) | RSD (%) | Average recovery (%) RSD (%) | |
| Salvianic acid A sodium | 0.29 | 1.90 | 1.51 | 102.66 | 3.40 |
| Rosmarinic acid | 0.59 | 1.44 | 1.29 | 103.02 | 3.85 |
| Salvianolic acid B | 0.50 | 0.29 | 1.87 | 100.19 | 1.15 |
| Dihydrotanshinone I | 0.39 | 1.09 | 0.22 | 98.38 | 0.23 |
| Cryptotanshinone | 0.30 | 1.98 | 0.54 | 98.16 | 2.91 |
| Tanshinone I | 0.44 | 1.21 | 1.70 | 96.56 | 3.12 |
| Tanshinone IIA | 0.44 | 0.43 | 0.43 | 104.52 | 2.56 |

(Note: This table presents the methodological parameters of the developed HPLC-DAD method, indicating that this method is reliable for determining the active compounds in Danshen medicines.)

**Table S5: Comparison of chemometric models based on different pretreatments**

| Model | Wavelength (nm) | Pretreatments | *LV* | *R_c_^2^* | *SEC*  (%) | *SECV*  (%) | *R_p_^2^* | *SEP*  (%) | *RPD* |
| --- | --- | --- | --- | --- | --- | --- | --- | --- | --- |
| TA2 | 850-2500 | SNV; DT | 13 | 0.759 | 0.048 | 0.054 | 0.797 | 0.045 | 2.18 |
|  | 850-2500 | FD; SNV; DT | 12 | 0.826 | 0.039 | 0.046 | 0.892 | 0.047 | 2.09 |
|  | 850-2500 | SD; SNV; DT | 12 | 0.849 | 0.045 | 0.046 | 0.867 | 0.039 | 2.51 |
|  | 1100-2500 | SNV; DT | 15 | 0.800 | 0.045 | 0.049 | 0.808 | 0.036 | 2.72 |
|  | 1100-2500 | FD; SNV; DT | 12 | 0.830 | 0.039 | 0.046 | 0.896 | 0.029 | 3.38 |
|  | **1100-2500** | **SD; SNV; DT** | **12** | **0.933** | **0.023** | **0.029** | **0.932** | **0.025** | **3.92** |
| SAB | 850-2500 | SNV; DT | 9 | 0.710 | 0.949 | 0.994 | 0.722 | 1.104 | 1.61 |
|  | 850-2500 | FD; SNV; DT | 13 | 0.825 | 0.723 | 0.741 | 0.819 | 0.870 | 2.04 |
|  | 850-2500 | SD; SNV; DT | 14 | 0.877 | 0.461 | 0.562 | 0.860 | 0.774 | 2.29 |
|  | 1100-2500 | SNV; DT | 14 | 0.742 | 0.748 | 0.780 | 0.715 | 0.995 | 1.78 |
|  | **1100-2500** | **FD; SNV; DT** | **14** | **0.906** | **0.513** | **0.556** | **0.835** | **0.723** | **2.46** |
|  | 1100-2 500 | SD; SNV; DT | 11 | 0.817 | 0.548 | 0.650 | 0.863 | 0.960 | 1.85 |
| TTC | 850-2500 | SNV; DT | 11 | 0.817 | 0.079 | 0.087 | 0.801 | 0.112 | 1.46 |
|  | 850-2500 | FD; SNV; DT | 15 | 0.886 | 0.059 | 0.068 | 0.941 | 0.064 | 2.55 |
|  | 850-2500 | SD; SNV; DT | 14 | 0.944 | 0.043 | 0.045 | 0.941 | 0.040 | 4.08 |
|  | 1100-2500 | SNV; DT | 12 | 0.834 | 0.074 | 0.081 | 0.840 | 0.081 | 2.01 |
|  | 1100-2500 | FD; SNV; DT | 13 | 0.887 | 0.057 | 0.068 | 0.924 | 0.055 | 2.96 |
|  | **1100-2500** | **SD; SNV; DT** | **14** | **0.944** | **0.043** | **0.045** | **0.941** | **0.040** | **4.08** |

(Note: This table presents the results of different MPLS models regarding different band ranges and pretreatments. TA2, SAB and TTC are the abbreviations of tanshinone IIA, salvianolic acid B and the total of tanshinone IIA, tanshinone I and cryptotanshinone, respectively)

**
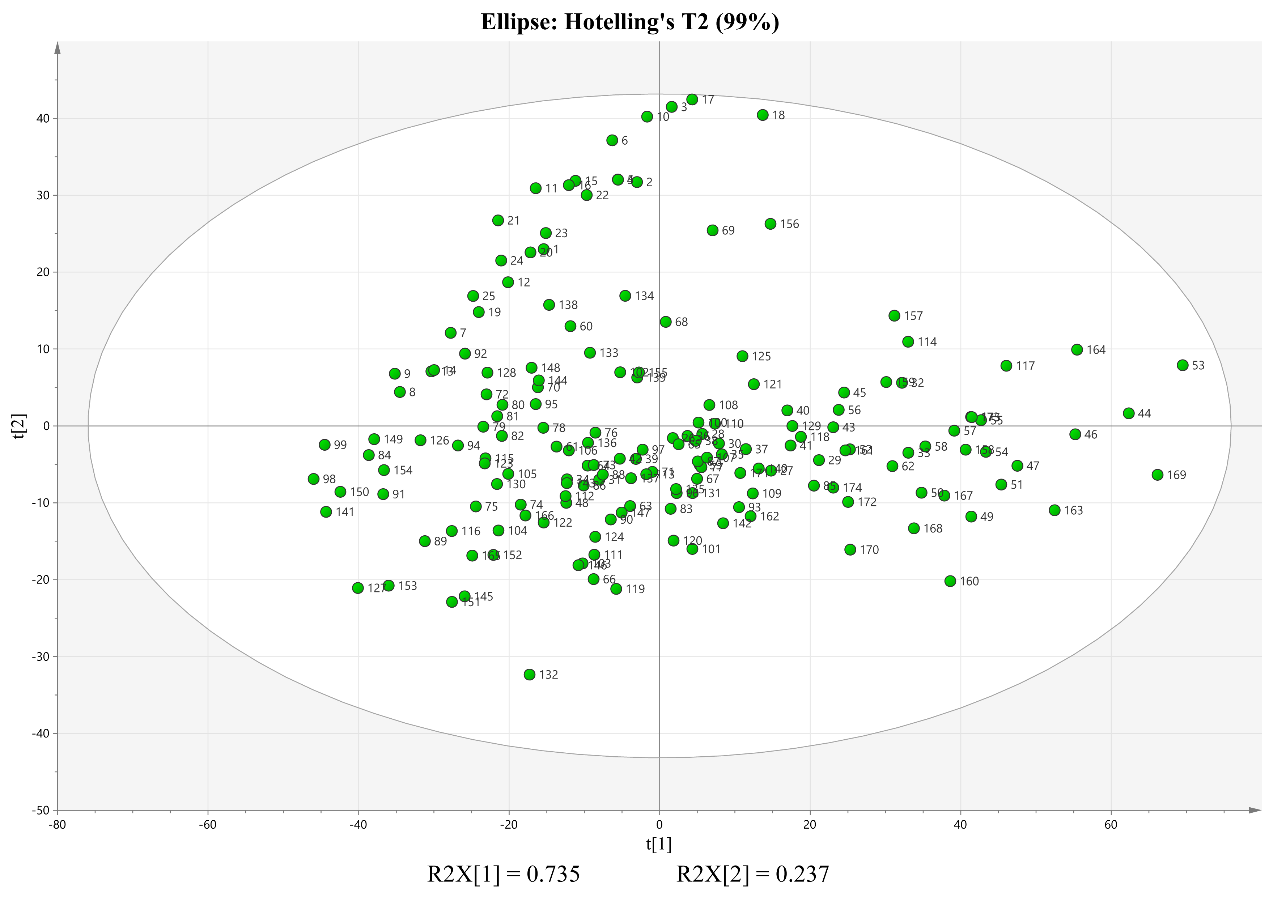
**

Figure S1: The 99% confidence ellipses for the outlier’s detection based on principal component analysis

(Note: This figure presents the 99% confidence ellipses for all used spectral data based on principal component analysis and indicates that all used spectra are qualified for next chemometric analysis.)
